# Impairment-targeted exercises for older adults with knee pain: a proof-of-principle study (TargET-Knee-Pain)

**DOI:** 10.1186/s12891-016-0899-9

**Published:** 2016-01-29

**Authors:** Laurence R. J. Wood, Milica Blagojevic-Bucknall, Siobhán Stynes, Deborah D’Cruz, Ricky Mullis, Rebecca Whittle, George Peat, Nadine E. Foster

**Affiliations:** Arthritis Research UK Primary Care Centre, Research Institute for Primary Care & Health Sciences, Keele University, Keele, Staffordshire ST5 5BG UK; General Practice and Primary Care Research Unit, Department of Public Health and Primary Care, University of Cambridge, Cambridge, CB1 8RN UK

**Keywords:** Knee joint, Exercise therapy, Postural balance, Muscle Weakness, Pain

## Abstract

**Background:**

Therapeutic exercise is an effective intervention for knee pain and osteoarthritis (OA) and should be individualised. In a preliminary, proof-of-principle study we sought to develop a home exercise programme targeted at specific physical impairments of weak quadriceps, reduced knee flexion range of motion (ROM) and poor balance, and evaluate whether receipt of this was associated with improvements in those impairments and in patient-reported outcomes among older adults with knee pain.

**Methods:**

This community-based study used a single group, before-after study design with 12-week follow-up. Participants were 58 adults aged over 56 years with knee pain and evidence of quadriceps weakness, loss of flexion ROM, or poor balance, recruited from an existing population-based, observational cohort. Participants received a 12-week home exercise programme, tailored to their physical impairments. The programme was led, monitored and progressed by a physiotherapist over six home visits, alternating with six telephone calls. Primary outcome measures were maximal isometric quadriceps strength, knee flexion ROM and timed single-leg standing balance, measured at baseline, 6 and 12 weeks by a research nurse blinded to the nature and content of participants’ exercise programmes. Secondary outcome measures included the WOMAC.

**Results:**

At 12 weeks, participants receiving strengthening exercises demonstrated a statistically significant change in quadriceps isometric strength compared to participants not receiving strengthening exercises: 3.9 KgF (95 % CI 0.1, 7.8). Changes in knee flexion ROM (2.1° (−2.3, 6.5)) and single-leg balance time (−2.4 s (−4.5, 6.7)) after stretching and balance retraining exercises respectively, were not found to be statistically significant. There were significant improvements in mean WOMAC Pain and Physical Function scores: −2.2 (−3.1, −1.2) and −5.1 (−7.8, −2.5).

**Conclusions:**

A 12-week impairment-targeted, home-based exercise programme for symptomatic knee OA appeared to be associated with modest improvements in self-reported pain and function but no strong evidence of greater improvement in the specific impairments targeted by each exercise package, with the possible exception of quadriceps strengthening.

**Trial registration:**

Clinical Trial Registration Number: ISRCTN 61638364

Date of registration: 24 June 2010

**Electronic supplementary material:**

The online version of this article (doi:10.1186/s12891-016-0899-9) contains supplementary material, which is available to authorized users.

## Background

Persistent knee pain, typically attributed to osteoarthritis (OA), affects an estimated 25 % of adults aged over 55 years [1]. The cumulative evidence from clinical trials conducted over the past 20 years is overwhelmingly in favour of the effectiveness of supervised exercise programmes in reducing knee pain and improving function [2] and exercise is now consistently recommended in national and international clinical guidelines as a core treatment [3–7]. The observed benefits of exercise are, on average, small-to-moderate [8]. Within this group-average effect, the response to exercise may vary considerably from one patient to another. However, there is no strong evidence from meta-analysis of trials that can reliably identify patient subgroups that will benefit most from exercise therapy [9]. The need to tailor exercise therapy to individual patients is well-recognised [4, 6]. but it is not clear how this ought to be operationalised in practice nor whether patient-reported outcomes are improved as a result.

Previous observational studies have shown that there are several impairments that occur reasonably frequently in knee OA patient populations, are simple-to-measure, potentially-reversible by exercise therapy, and associated with patient-reported outcomes (pain and disability) [10–17]. This might suggest that one approach to individualising exercise therapy could be to match and target exercises to patients’ particular combinations of physical impairments. Whilst many intervention studies have tested the effectiveness of exercise programmes that incorporate features of individualisation to patients’ presenting impairments, the way in which they do this is rarely detailed. None, to our knowledge, have specified the way in which specific impairments in strength, range of motion and balance have been identified and addressed. There are, to date, no trials of stratified care (subgrouping and matching them to exercise treatments) for patients with knee OA. Research has proposed a preliminary clinical prediction rule to identify the patients who may not benefit from exercise but this has yet to be validated in external samples [18]. To investigate this further, and to inform the decision and design of a future clinical trial, we undertook a uncontrolled before-after study (TargET-Knee-Pain) to test the principle that exercises targeted at three specific physical impairments common in older adults with knee pain can significantly improve those impairments. We chose to focus on weak quadriceps muscles, a loss of range of knee joint flexion and poor balance. Each can be measured using simple methods (and hence could be practicable in routine primary care, the proposed setting for future trials and implementation). In addition to previous studies linking these impairments to patient-reported outcomes [10–13, 15–17] we had previously shown these impairments to be independently associated with patient-reported outcomes in our patient population [14] - A secondary aim was to establish to what degree any improvements in these factors may be reflected in improvements in self-reported knee pain, stiffness, and functional limitation.

## Methods

### Design overview

There are no general recipes for proof-of-principle studies but their purpose is to identify an efficacy signal for a planned intervention [19]. This study had a single-group, before-after design. Adults aged 56 years and over with knee pain and evidence of impaired knee flexion range of motion, quadriceps strength, or standing balance, were offered a 12-week tailored home exercise programme targeted to their impairments, and which included six supervised sessions in their home and six telephone calls to monitor their progress. Full details of the design and methods are available from the published study protocol [20].

### Setting and participants

Participants were recruited from the 6-year follow-up research clinic visits for a population-based observational cohort study of knee pain/osteoarthritis - the Clinical Assessment Study of the Knee (CAS(K)) [21, 22]. CAS(K) cohort participants were originally included recruited from the registers of three general practices in North Staffordshire between 2002 and 2003, irrespective of whether they had consulted for knee pain/OA. All were aged 50 years and over and reported knee pain within the previous 12 months. The inclusion criteria at 6-year follow-up for eligibility for the TargET-Knee-Pain intervention study were: one or more of the three target impairments below age- and gender-stratified threshold (thresholds based on the lowest quartile values for measurements taken at the baseline CAS(K) research clinic; Additional file 1); willing and able to commit to a programme of exercises for a 12-week period. Exclusion criteria were: total knee replacement of either knee joint; an existing diagnosis of inflammatory arthropathy; lower limb weakness from neurological conditions; receiving medication that adversely affects standing balance; open wounds on the anterior aspect of either distal shin; a self-report of unstable angina or uncontrolled hypertension/hypotension; an inner ear problem that compromises standing balance; no mobile or home telephone; unavailability for fortnightly home visits or telephone contact for the whole of a given working week of their potential involvement in the study; an inability to transfer independently from lying to sitting or from sitting to standing; currently receiving physiotherapy for their knee problem. The setting for the exercise programme was the participant’s home.

### Interventions

Participants received one or more of three home-based exercise packages (one for each of the three target physical impairments), dependent on which of the impairments they had. These packages were developed with reference to published literature and each exercise was systematically progressed through photographically-illustrated stages. Full details of each exercise package, including the photographically-iilustrated stages, are provided in the additional files published with the study protocol [20].

Strengthening exercises consisted of a series of resisted isometric and isotonic quadriceps contractions, using a combination of body weight resistance and rubber exercise bands. Stretching exercises involved prolonged end-range knee flexion stretches in various positions, utilising body weight or manual overpressure. Balance retraining exercises were a series of static and dynamic activities designed to progressively challenge participants’ balance reactions. They included activities, such as balancing in positions with additional balance perturbation in the form of upper limb activities (e.g. ball bouncing and catching) and forward and backward straight-line-walking in various gait patterns (e.g. tip-toe and heel-to-toe). Exercises were targeted at the knee with below-threshold impairment where possible and were selected and the level-of-difficulty tailored to the abilities of the participant based on an assessment by a study physiotherapist during the first physiotherapist home visit. Exercises were performed bilaterally in those with bilateral impairment. Exercises were performed at least daily. Monitoring of participants’ progress and appropriate progression of exercises was achieved through fortnightly physiotherapist home visits, alternating with fortnightly telephone calls over the 12 week follow-up period.

Balance exercises were progressed according to ability. Once a participant could hold a position for 30 s they were progressed to the next level, or they repeated the same exercise but with a more challenging foot position (Additional file 2). For strengthening exercises participants progressed to the next level in dynamic exercises when they achieved the current level comfortably with no signs of fatigue or pain. For the theraband exercises they were progressed once they could complete two sets of 10–12 good quality repetitions slowly without signs of fatigue or pain on three consecutive days (Additional file 2). For ROM exercises the exercises were assigned according to an optimum starting position that the participant could comfortably achieve and they were advised to hold the position of stretch for up to 30 s. They were generally given up to three to five different stretches.

Adherence was encouraged by the use of progress charts and daily exercise diaries.

### Outcomes and follow-up

Outcome measures were administered by a study nurse, independent of the study physiotherapists and blinded to the particular impairment(s) and, hence, the exercise package(s) participants were receiving. Measures were taken at the first baseline nurse home visit, and again at the second and third nurse visits at weeks 6 and 12.

The three primary outcome measures were degree of active end-range knee flexion, measured with a 12-inch universal goniometer in supine; maximal isometric quadriceps strengths at 90° of knee flexion, measured with a Chattillon DFX-200 electronic dynamometer, and a modified version of Franchignoni et al’s timed standing balance test (single-leg stance, hands on hips, up to a maximum of 30 s) [23, 24]. Intra-class correlation coefficients for intra-observer reliability for similar measures were previously estimated at 0.67–0.85 [24].

Secondary outcome measures included self-report measures of pain, stiffness and physical function (WOMAC LK 3.1) [25], and self-report measures of the frequency of knee symptoms [26], perceived ‘bothersomeness’[27] and global change in the knee problem [28]. Adherence to the exercise programme was evaluated with one closed question at 6 and 12 weeks, [29] and one open question with free-text response at 12 weeks. Barriers to adherence, the acceptability and ways to improve the programme were evaluated by a combination of closed and open questions at 12 weeks (Additional file 3).

### Statistical analysis

Power calculations, based on observed effect sizes in previous trials of exercises for patients with knee OA, suggested that a sample size of 60 individuals would be capable of detecting an 8° improvement in the degree of knee flexion or an 8 Kg improvement in quadriceps strength with approximately 86 % power, given a Type I error rate of 5 %.

Descriptive characteristics of study completers were compared with individuals who were found to be eligible at the CAS(K) 6-year follow-up research clinic visit but who subsequently refused either the nurse call or the offer of intervention. The comparison used information collected at CAS(K) 6-year follow-up on age, sex, educational attainment, perceived financial strain [30], social networks [31], body mass index, Hospital Anxiety and Depression scale (HADS [32]), SF-12 [33], knee flexion ROM, quadriceps isometric strength, single-leg standing balance time and WOMAC.

The relationships between being allocated impairment-specific treatment package and primary outcome measures were assessed by fitting linear regression models to each of the three primary outcome impairment measures, at 6 weeks and 12 weeks, adjusting for baseline score. This was done separately for each exercise package (e.g. those allocated strengthening exercises vs not allocated strengthening exercises) and adjusting for age, gender, and allocation to other impairment-specific treatment packages. The estimate for quadriceps isometric strength was also adjusted for body mass index (BMI). These main findings were explored further by: (a) describing levels of impairment among study completers, using their previous measurements from CAS(K) (baseline, 3- and 6-year follow-up), thereby placing within-group change in a longer-term prior trajectory; (b) estimating the percentage of study completers attaining age-gender stratified normative values for each impairment at 12 weeks, thereby seeking to explore potential ceiling effects [34–36]; (c) excluding participants with inadequate adherence (defined as not having done any of the exercises at all for 7 consecutive days or for a cumulative total of 12 days throughout the 12-week study period).

All study completers were combined for the secondary outcome analyses. Paired t-tests (or non-parametric equivalents) were used to test the changes in WOMAC Pain, Stiffness, and Physical Functioning subscale scores between baseline and 12 weeks. Secondary outcomes were further explored by: (d) repeating the main analyses after excluding participants with inadequate adherence; (e) describing prior WOMAC scores from CAS(K) baseline, 3- and 6-year follow-up; (f) examining correlations between changes in impairments and changes in WOMAC Pain, Stiffness and Physical Function scores; (g) comparing average changes in WOMAC Pain, Stiffness and Physical Function scores in participants receiving two or more exercise packages to those receiving only one exercise package, adjusting for those covariates associated with change in relevant WOMAC score and group membership. Descriptive frequencies were used to summarise the remaining secondary outcome measures. Thematic analysis was used to extract key themes on barriers to adherence and acceptability of the intervention from responses to open-ended questions.

## Results

**Fig. 1:**
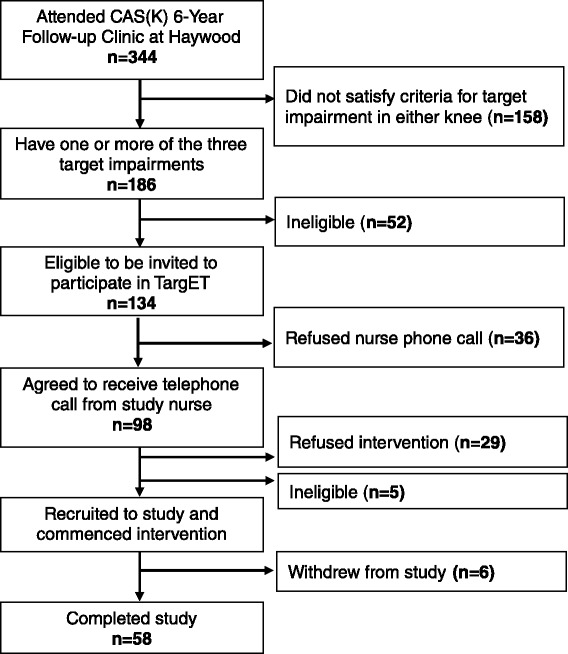
Flow of participants

Between May 2009 and January 2010, of 344 adults attending the CAS(K) 6-year follow-up research clinics, 134 (39 %) were eligible to participate in the TargET-Knee-Pain study. Sixty-four (48 %) were recruited into the study and gave written informed consent to participate and provided baseline questionnaire and impairment measures data. Fifty-eight completed the study (Fig. 1).

The 58 study completers had a mean age of 68.7 (SD 7.8) years. 62 % were female and 95 % were overweight or obese (BMI ≥ 25 kg/m^2^). From the CAS(K) 6-year radiographs, 50 (86 %) had structural changes in at least one knee, consistent with definite osteoarthritic changes (Kellgren & Lawrence Grade ≥ 2 in tibiofemoral or patellofemoral joint [37]). Study completers were similar to the 65 individuals who were found eligible at the clinic visit but who subsequently refused either the nurse call (*n* = 36) or the offer of intervention (*n* = 29), although they did appear to have more extensive social networks (Table 1).

**Table 1:** Descriptive characteristics of study completers and refusals

	Study completers	Refused nurse call or offer of intervention
	(*n* = 58)	(*n* = 65)
Age (years)	68.7 (7.8)	69.8 (6.9)
Female gender, n (%)	36 (62 %)	35 (54 %)
Educational attainment: school age only, n (%)	48 (84 %)	51 (84 %)
Lower social network index^a^, n (%)	23 (42 %)	40 (68 %)
Body mass index (kg/m^2^)	32.1 (6.2)	31.6 (5.6)
HAD^b^ Anxiety score > 8, n (%)	14 (24 %)	17 (27 %)
HAD Depression score > 8, n (%)	8 (14 %)	6 (9 %)
Perceived financial strain^c^, n (%)	25 (43 %)	24 (37 %)
SF12: PCS (0–100)	34.4 (8.2)	35.8 (11.3)
SF12: MCS (0–100)	50.4 (10.2)	50.2 (10.1)
Knee flexion ROM (degrees)	126.3 (12.1)	124.8 (15.3)
Quadriceps isometric strength (KgF)	20.8 (7.5)	18.8 (7.7)
Single-leg standing balance time (seconds): median (IQR)	5.0 (9.6)	4.8 (11.8)
WOMAC Pain (0–20)	7.5 (3.7)	6.8 (4.3)
WOMAC Stiffness (0–8)	3.5 (1.6)	3.3 (2.1)
WOMAC Physical Function (0–68)	25.9 (12.8)	26.8 (14.5)

Figures are mean (standard deviation) unless otherwise stated

^a^Classed as low or medium on Berkman-Syme Social Network Index [31]

^b^Hospital Anxiety & Depression scale [32]

^c^Responded “find it a strain” or “have to be careful” in response to single item on perceived adequacy of income [30]

**Fig. 2:**
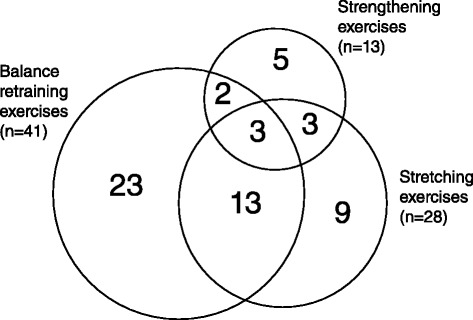
Area-proportional Venn diagram of numbers of individuals receiving exercises targeted at each of the three impairment

Thirty-seven completers (64 %) had only one of the target physical impairments, 18 (31 %) had two impairments, and 3 (5 %) had all three (Fig. 2). 28 completers had impaired flexion range of motion in at least one of their knees and so received exercises targeted at this impairment; 13 had impaired quadriceps strength and received quadriceps-strengthening exercises, and 41 had impaired single-leg standing balance, requiring balance-retraining exercises.

### Primary outcomes

There were no statistically significant differences in knee flexion ROM or single-leg standing balance between those receiving the impairment-targeted exercise programme and those who did not at either 6 or 12 weeks (Table 2). At 6 weeks, there was no statistically significant difference in quadriceps isometric strength between the groups, but at 12 weeks, quadriceps isometric strength was 3.9KgF (95 % CI: 0.1, 7.8) higher in those who receiving strengthening exercises compared to those not receiving the strengthening exercises.

**Table 2:** Primary outcomes, by allocation to each impairment-specific exercise package in turn

	Allocated impairment-specific exercise package	Not allocated impairment-specific exercise package	Coefficient (95 % CI)^a^	*p*-value
	Stretching exercises			
	*n* = 28	*n* = 30		
Knee flexion range of motion (degrees): mean (SD)				
Baseline	113.4 (10.6)	132.6 (7.1)		
6 weeks	119.4 (9.3)	131.9 (7.1)	2.06 (−1.36, 5.48)	0.232
12 weeks	123.1 (8.5)	131.2 (6.3)	2.12 (−2.28, 6.51)	0.338
	Strengthening exercises			
	*n* = 13	*n* = 45		
Quadriceps isometric strength (KgF): mean (SD)				
Baseline	12.7 (8.9)	18.9 (8.9)		
6 weeks	17.1 (8.1)	20.5 (8.8)	0.02 (−4.15, 4.20)^b^	0.992
12 weeks	21.9 (6.1)	20.6 (8.4)	3.94 (0.12, 7.77)^b^	0.043
	Balance retraining exercises			
	*n* = 41	*n* = 17		
Single-leg standing balance time (seconds): median (IQR)				
Baseline	3.0 (3.5)	14.5 (14.5)		
6 weeks	5.0 (15.0)	17.5 (13.0)	−1.6 (−8.4, 5.1)	0.633
12 weeks	9.0 (23.3)	21.5 (17.3)	−1.5 (−9.3, 6.4)	0.713

^a^Values are coefficients (95 % confidence intervals) from linear regression models of allocated impairment-specific exercise package vs. not allocated impairment-specific exercise package for primary outcomes at 6 weeks and 12 weeks, adjusted for baseline score, age, gender, and concurrent allocation to other impairment-specific exercise packages

^b^Coefficients additional adjusted for body mass index

Observed improvements in the impairments over the 12 week study period in those receiving each of the exercise packages contrasted with progressive deterioration in those impairments among the same individuals over the 6 years prior to the start of the current study (Table 3). The proportion of participants receiving each exercise package that attained age-gender normative values at 12 weeks in the target impairment was 27/28 (96 %) for knee flexion ROM; 6/13 (46 %) for quadriceps strength, and 17/41 (41 %) for single-leg standing balance. Compared with all participants receiving each exercise package, those who were adherent with the exercises had higher within-group mean changes between baseline and 12 weeks for all three impairments: knee ROM 11.3 vs 9.6°; quadriceps strength 11.8 vs 9.3 KgF; single-leg standing balance 10.2 vs 8.4 s. The study nurse was unblinded at one or more of the three time points in 18 cases; exclusion of these cases did not alter the results.

**Table 3:** Primary and secondary outcome measures during the TargET intervention period and in the preceding 6 year CAS(K) observational period

			CAS(K) (Observational period)					TargET (Intervention period)		
		N	Baseline	18 mo	36 mo	54 mo	72 mo	Baseline	6 wk	12 wk
Knee flexion ROM (degrees)	Mean (SD)	28	123.7 (14.1)	-	119.8(15.6)	-	114.8(11.6)	113.4(10.6)	119.4(9.3)	123.1(8.5)
Quadriceps isometric strength (KgF)	Mean (SD)	13	16.3(9.5)	-	15.4 (8.4)	-	11.2(5.7)	12.7(8.9)	17.1(8.1)	21.9(6.1)
Single-leg standing balance time (sec)	Median (IQR)	41	6.0(9.0)	-	3.5(8.0)	-	2.0 (1.0)	3.0(3.75)	5.0(14.5)	9.0(23.25)
WOMAC Pain (0–20)	Mean (SD)	58	5.6(4.3)	6.4 (4.2)	7.5(3.9)	7.5(4.2)	7.7(3.8)	7.5(3.7)	5.7(3,7)	5.3(3.4)
WOMAC Stiffness (0–8)	Mean (SD)	58	2.7 (1.9)	3.0(1.8)	3.2(2.0)	3.5(1.9)	3.7(1.5)	3.5(1.6)	2.9(1.9)	2.7(1.9)
WOMAC Physical Function (0–68)	Mean (SD)	58	19.3(15.0)	22.5(15.1)	23.2(13.8)	25.6(15.4)	27.4(13.3)	25.9(12.8)	22.1(13.5)	20.7(13.0)

### Secondary outcomes

Statistically significant improvements from baseline to 12 weeks were observed for Pain (mean change −2.2; 95 % CI −3.1, −1.2), Stiffness (−0.8; −1.2, −0.3), and Physical Function (−5.1; −7.8, −2.5) (Table 4). Improvements were greater when the analyses were restricted to those who were adherent to the exercises (Pain (−3.1; −4.2, −2.0), Stiffness (−1.1; −1.6, −0.5), and Physical Function (−6.9; −9.9, −3.9)).

**Table 4:** WOMAC secondary outcomes, all 58 study completers combined

WOMAC Pain (0–20), mean (SD)	
Baseline	7.5 (3.7)
12 weeks	5.3 (3.4)
Baseline-12 weeks change, mean (95 % CI)	−2.2 (−3.1, −1.2)
WOMAC Stiffness (0–8), mean (SD)	
Baseline	3.5 (1.6)
12 weeks	2.7 (1.9)
Baseline-12 weeks change, mean (95 % CI)	−0.8 (−1.2, −0.3)
WOMAC Physical Function (0–68), mean (SD)	
Baseline	25.9 (12.8)
12 weeks	20.7 (13.0)
Baseline-12 weeks change, mean (95 % CI)	−5.1 (−7.8, −2.5)

As seen with the primary outcomes, improvements in WOMAC followed observed-worsening over the 6 years prior to intervention (Table 3). Correlation analyses revealed that improvements in knee flexion ROM from baseline to 12 weeks were associated with reductions in WOMAC Physical Function scores over this time period (Pearson’s correlation coefficient (r) = −0.285, *p*-value = 0.030) and that improvements in balance were associated with reductions in WOMAC Stiffness scores over this same time period (r = −0.266, *p*-value = 0.046). All other associations (*n* = 7) were in a similar direction but failed to reach statistical significance. Those receiving two or three exercise packages had larger improvements in all WOMAC scores from baseline to 12 weeks, compared with those receiving only one (within-group mean changes: Pain −3.3 (−5.1, −1.6) vs −1.5 (−2.6, −0.3); Stiffness −1.2 (−1.9, −0.6) vs −0.5 (−1.1, 0.2); Physical Function −9.7 (−14.6, −4.8) vs −2.6 (−5.5, 0.4)). Physical Function between-group mean difference was the only statistically significant finding (7.1 (1.9, 12.4)).

At 12 weeks, 23 (40 %) reported pain on most or all days (vs 37 (64 %) at baseline); 5 (9 %) reported their knee problem as “very much” or “extremely” bothersome (vs 20 (35 %) at baseline), and 78 % reported their knee problem was “better”, “much better” or “completely recovered” (Table 5).

**Table 5:** Other secondary outcomes, all 58 study completers combined

	Baseline		12 weeks	
Frequency of knee symptoms, n *(%)* ^a^				
No days	1	*(2)*	4	*(7)*
Few days	7	*(12)*	16	*(27)*
Some days	12	*(21)*	15	*(26)*
Most days	18	*(31)*	19	*(33)*
All days	19	*(33)*	4	*(7)*
Bothersomeness of knee problem, n *(%)* ^a^				
Not at all	2	*(3)*	10	*(17)*
Slightly	11	*(19)*	20	*(34)*
Moderately	24	*(41)*	23	*(40)*
Very much	15	*(26)*	4	*(7)*
Extremely	5	*(9)*	1	*(2)*
Patient global rating of change, n *(%)*				
Completely recovered	-		1	*(2)*
Much better	-		18	*(31)*
Better	-		26	*(45)*
No change	-		10	*(17)*
Worse	-		0	*(−)*
Much worse	-		3	*(5)*

^a^57 completed the item at baseline

There were no adverse events recorded for any participant during their 12 week exercise programme. The exercises were generally well-tolerated by participants; nine gave examples of what could have been done to make them more willing to practise them, and seven gave examples of where their overall experience of participating in the study could have been improved.

## Discussion

This proof-of-principle study found that a relatively simple 12-week impairment-targeted, home-based exercise programme for symptomatic knee OA appeared to be well-tolerated, and that in uncontrolled before-after comparisons individuals undertaking this programme reported modest improvements in pain and functional limitation. After adjusting for baseline values and other selected potential confounders, there was, however, no strong evidence of greater improvement within the 12-week timeframe in the specific impairments targeted by each exercise package, with the possible exception of improved quadriceps strength in those allocated to the strengthening exercise package.

The choice of sampling frame – a well-characterised, community-based cohort of older adults with a history of knee pain and varying degrees of structural OA changes – was convenient, efficient, and provided the advantage of prior measurements: a feature rarely available in intervention studies. These prior measurements provided reassurance that the observed changes in the study are not explained wholly by regression to the mean. The sampling frame also imposed some constraints. It resulted in less severe cases than those consulting with knee pain (TargET mean WOMAC Pain score was 7.5 compared with 9.1 in two previous trials of knee pain in primary care consulters [29, 38]). The sampling frame provided a fixed pool of potentially eligible participants, sufficient to achieve our recruitment target of 60 individuals, but without the scope to select an equal number of participants with each physical impairment, or to balance combinations of the impairments. The relative frequencies of impaired range of motion (48 %), strength (22 %) and balance (71 %) in the 58 study completers were similar to those seen in the 134 eligible participants (49 %, 29 %, and 66 % respectively), suggesting no strong evidence of selective over- or under-recruitment. Combinations of impairments and potential carry-over effects of one type of exercise package on other impairments limit the extent to which observed changes in the impairments can be attributed to specific exercise packages although we did try to adjust for these co-interventions in the analysis. Since we wanted to test the proof-of-principle that targeted exercises would lead to improvements in specific impairments, we wanted to maximise adherence to the exercise programmes used in this study, which were, therefore, relatively intensive, in terms of one-to-one participant-to-physiotherapist contact. Current physiotherapy practice in the UK averages 4–5 treatment sessions for a typical patient with clinical knee OA [39].

The observed changes can be viewed in the context of changes seen in previous studies of exercise for knee pain and osteoarthritis. A systematic review of exercise for lower limb osteoarthritis found the most effective approach to involve combining exercises to increase strength, flexibility and aerobic capacity [2]. The most recent systematic review of exercise for knee osteoarthritis estimated that the best estimates of the immediate post-treatment effects were absolute reduction in pain and self-reported functional limitation scores of 12 % and 10 % respectively [8]. The 2.2 and 5.1 mean reductions in WOMAC Pain and Function subscales observed in our study are similar to these (absolute change of 11 % and 7.5 % respectively). McCarthy et al [40]. observed modest reductions in functional limitations and pain at eight weeks with home exercises prescribed and then progressed at four weeks in the clinic setting, with the addition of 16 twice-weekly group exercise classes providing further reductions in both pain and functional difficulties (WOMAC Pain and Function subscale change scores of 2.1 and 6.0 points, respectively). Changes of similar magnitude were observed in the UK-based APEX [38] and ESCAPE [41] trials, whose interventions, respectively, involved a median of six individual advice and exercise sessions, and 12 class-based sessions of rehabilitation, combining exercise and self-management education.

The mean change in muscle strength in the current study compares favourably with changes observed in Lange et al.’s [42] systematic review (mean change across the studies in the review = +17.4 % (range −10.5 to +49.5 %)). However, our results need to be treated with caution, since they are based on a sample of only 13 participants who undertook the quadriceps strengthening exercises, and so could be a function of the relatively short duration of the current study, since gains from exercising are lost over time unless patients are actively encouraged to continue exercising [43]. Reviews of the effectiveness of T’ai Chi suggest that it improves both static and dynamic balance in older people [44–46] and can provide improvements in pain and physical function for patients with knee OA [47, 48]. A systematic review of exercises to improve balance in older people (gait, balance, coordination and functional tasks) found pooled mean differences in single-leg standing balance of 3.13 s for single exercises and 5.03 s for multiple-type exercises [49]. We could find no systematic reviews of the effects of stretching on reduced knee ROM in similar populations to ours, although the prescription of stretching exercises to improve joint ROM is well-recognised as good practice, according to international guidelines [50].

### Study limitations

The study is limited by the small sample size and short-term outcomes, but improvements were shown in the context of prior long-term deterioration. The before-after design and lack of a control group mean that this study must be interpreted as an initial evaluation of the principle of impairment-targeted exercise, rather than as providing evidence of the comparative clinical effectiveness of this approach. Our study used simple, single measures of each impairment but recommended core sets of standardised measures for impairments that have recently been reported, for example standing balance [51], may be preferable since they offer a common basis across researchers and practitioners, and the potential to capture these constructs more completely and with less error.

## Conclusions

Targeting exercises according to individuals’ physical impairments, such as weak quadriceps, loss of range-of-motion and poor balance, appears to be a safe and well-tolerated approach to tailoring non-pharmacological treatment for people with knee pain and osteoarthritis and could be applied to treatable physical impairments other than those studied here [52]. We found no strong evidence to suggest a specific effect of the exercise packages targeting loss of knee flexion range of motion and single-leg standing balance at 12 weeks. However, improvements in all targeted impairments and in self-reported pain and function during the intervention period were observed after prolonged deteriorations in the preceding 6 years. The effectiveness of specific, impairment-targeted exercise, compared with other approaches to exercise, cannot be ascertained from this single-group, proof-of-principle study and could be the focus of future research.

## Declarations

### Ethics approvals and consent to participate

Written informed consent to take part in this study was obtained in person from eligible participants. The study was approved by West Midlands – Black Country Research Ethics Committee (REC reference: 08/H1202/179).

### Availability of data and materials

Consent to deposit clinical data on a publicly accessible repository was not obtained from participants before this study began. The Centre has established data sharing arrangements to support joint publications and other research collaborations. Applications for access to anonymised data from our research databases are reviewed by the Centre’s Data Custodian and Academic Proposal (DCAP) Committee and a decision regarding access to the data is made subject to the NRES ethical approval first provided for the study and to new analysis being proposed. Further information on our data sharing procedures can be found on the Centre’s website (http://www.keele.ac.uk/pchs/publications/datasharingresources/) or by emailing the Centre’s data manager (primarycare.datasharing@keele.ac.uk).

## Additional files

Additional file 1:
**Age and gender thresholds for study inclusion.** Table providing information on the specific measurement thresholds used to determine inclusion and allocation of specific exercise packages. (DOCX 33 kb) Additional file 2:
**Progression of exercises.** Tables showing the scheme used to progress exercises in each of the three exercise packages. (DOC 51 kb) Additional file 3:
**Question content and response options of secondary outcome measures.** Format of individual questions used to measure secondary outcomes. (DOCX 14 kb)

## Abbreviations

CAS(K): clinical assessment study of the knee
CI: confidence interval
HADS: hospital anxiety and depression scale
IQR: inter-quartile range
MCS: mental component score
OA: osteoarthritis
PCS: physical component score
ROM: range of motion
WOMAC: Western Ontario & McMaster Universities Osteoarthritis Index
