# Supplementary material for: Impairment-targeted exercises for older adults with knee pain: a proof-of-principle study (TargET-Knee-Pain)
Source: BMC Musculoskelet Disord. 2016 Jan 29;17:47. doi: 10.1186/s12891-016-0899-9 (PMC4731955; doi:10.1186/s12891-016-0899-9)
Supplement: Additional file 1: — Age and gender thresholds for study inclusion. Table providing information on the specific measurement thresholds used to determine inclusion and allocation of specific exercise packages. (DOCX 33 kb) [file 12891_2016_899_MOESM1_ESM.docx]

**Additional file 1. Age and gender thresholds for study inclusion**

|  | Male | Female |
| --- | --- | --- |
| Range of knee joint flexion (degrees) |  |  |
| 50-64 years | < 128° | < 127° |
| 65-74 years | < 125° | < 122° |
| 75+ years | < 120° | < 117° |
| Isometric quadriceps strength (kilograms force) |  |  |
| 50-64 years | < 18.1 | < 11.3 |
| 65-74 years | < 17.2 | < 9.4 |
| 75+ years | < 13.9 | < 9.0 |
| Single-leg standing balance (seconds) |  |  |
| 50-64 years | < 8 | < 5 |
| 65-74 years | < 3 | < 3 |
| 75+ years | < 2 | < 2 |

Reproduced from: Wood et al. BMC Musculoskeletal Disorders 2011, 12:2 http://www.biomedcentral.com/1471-2474/12/2
