# Supplementary material for: Impairment-targeted exercises for older adults with knee pain: a proof-of-principle study (TargET-Knee-Pain)
Source: BMC Musculoskelet Disord. 2016 Jan 29;17:47. doi: 10.1186/s12891-016-0899-9 (PMC4731955; doi:10.1186/s12891-016-0899-9)
Supplement: Additional file 2: — Progression of exercises. Tables showing the scheme used to progress exercises in each of the three exercise packages. (DOC 51 kb) [file 12891_2016_899_MOESM2_ESM.doc]

**Balance exercises**

| **STANCE** | | LEVEL 1 | | LEVEL 2 | | LEVEL 3 | | LEVEL 4 | | LEVEL 5 |
| --- | --- | --- | --- | --- | --- | --- | --- | --- | --- | --- |
| **Feet together** | A Stand unsupported 30 seconds B Stand with hands on hips  30 seconds | | **A**  Stand with hands raised parallel with shoulders  30 seconds | | A Stand with one eye closed  30 seconds B Stand and throw small ball against wall  1x10 reps | | A Stand on a cushion and close one eye  30 seconds B Stand on a cushion and throw ball against wall  30 secs | |  | |
| **SEMI TANDEM** | | C Stand lightly holding support  30 seconds | | B Stand unsupported  30 seconds  **C**  Stand with hands on hips  30 seconds | | **C**  Stand with hands raised parallel with shoulders  30 seconds | | C Stand with one eye closed  30 seconds D Stand and throw ball against wall  30 seconds | | A/B Repeat level 4 standing on a cushion |
| TANDEM STAND | | D Stand lightly holding support  30 seconds | | D Stand unsupported  30 seconds  Stand with hands on hips  30 seconds | | D Transfer weight from front to back foot  1x10 reps E Stand with hands raised parallel with shoulders  30 seconds | | E Stand with one eye closed  30 seconds | | C Stand on a cushion and throw small  ball against wall  1x10 reps |
| **ONE LEG STAND** | |  | | E Hold support  30 seconds | | F No support  30 seconds  Stand with hands on hips  30 seconds | | F Stand with hands raised parallel with shoulders  30 seconds | | D Stand on a cushion with one eye closed  30 secs   E Stand on a cushion and throw ball against wall  x10 |
| **HEEL WALK** | |  | |  | | G Hold support  10 steps x 4 repetitions | | G No support  10 steps x 4 repetitions | |  |
| **TOE WALK** | |  | |  | | H Hold support  10 steps x 4 repetitions | | H No support  10 steps x 4 repetitions | |  |
| **TANDEM WALK/ HEEL-TOE WALK** | |  | |  | | i Hold support  10 steps x 4 repetitions Walk forward and backwards | | **i**  No support  10 steps  Walk forward and backwards | | F No support  10 steps x 4 repetitions  Walk forward and backwards |

**Strength exercises**

| **Exercise** | Level 1 | Level 2 | Level 3 | Level 4 |
| --- | --- | --- | --- | --- |
| **Double knee bend both legs/ mini squat** | A Hold Support  1 x 10 repetitions | A No support  1x10 repetitions |  |  |
| Forward Lunge | B Hold support, keeping back foot on ground  1 x 10 repetitions | B No support, keeping back foot on ground  1 x 10 repetitions | A Hold support, lifting back leg from ground  1 x 10 repetitions | A No support, lifting back leg from ground  1 x 10 repetitions |
| **Single Knee Bend one leg / mini squat** |  | C Hold support  1x10 repetitions | B No support  1x10 repetitions | B On step  1 x 10 repetitions |
| Wall sit/ slide |  | D Hold for up to  20 seconds | C Hold for up to  40 seconds | C Hold for up to  60 seconds |
| **In sitting, hold knee in fully straight position** | C Hold for up to  30 seconds | E Hold for up to  60 seconds | D Hold for up to  2 minutes |  |
| **Squat** |  |  |  | D Full squat x 10 |
| **Step up/ down slowly** |  | F Hold support, lead with affected leg  Repeat for 30 seconds | E No support, lead with affected leg  Repeat for 30 seconds | E No support, lead with affected leg  Repeat for 60 seconds |
| **Sit to Stand** | D Stand up slowly from arm chair using both hands  1x10 times | G Stand up slowly from arm chair using one hand  1x10 times | F Stand up slowly from chair without using your hands  1x10 times | F Stand up slowly from chair without using your hands  2 x10 times |
| *** Knee Straightening in sitting from 90-0 degrees extension** |  | H Yellow Theraband  1 x10 repetitions | G Red Theraband  1 x10 repetitions | G Green Theraband  1 x10 repetitions  (blue/black) |

*Progress to next level in dynamic exercises when participant can complete two sets of 10-12 good quality repetitions slowly without signs of fatigue or pain on three consecutive days
