# Supplementary material for: Impairment-targeted exercises for older adults with knee pain: a proof-of-principle study (TargET-Knee-Pain)
Source: BMC Musculoskelet Disord. 2016 Jan 29;17:47. doi: 10.1186/s12891-016-0899-9 (PMC4731955; doi:10.1186/s12891-016-0899-9)
Supplement: Additional file 3: — Question content and response options of secondary outcome measures. Format of individual questions used to measure secondary outcomes. (DOCX 14 kb) [file 12891_2016_899_MOESM3_ESM.docx]

**Additional file 3. Question content and response options of secondary outcome measures**

**Frequency of knee symptoms**

In the last **4 weeks** have you had pain **or** aching **or** stiffness in your knees? *[response options: no days, few days, some days, most days, all days]*

**Bothersomeness**

In the **last 2 weeks** how bothersome has your knee problem been? *[response options: not at all, slightly, moderately, very much, extremely]*

**Patient Global Rating of Change**

How do you think your knee problem has **changed** since you were **last visited** by the study nurse six weeks ago? *[response options: completed recovered, much better, better, no change, worse, much worse]*

**Adherence**

To what extent would you agree with the following statement? “In the last six weeks I have been doing the exercises as often as I was advised to” *[response options: strongly agree, agree, not sure, disagree, strongly disagree]*

**Non-validated questions on barriers to adherence, the acceptability and ways to improve the programme**

How did you find “getting round to doing” the exercises? *[response options: Very easy, Fairly easy, Neither easy nor difficult, Somewhat difficult, Very difficult]*

In general, how did you find the exercises themselves? *[response options: Very easy, Fairly easy, Neither easy nor difficult, Somewhat difficult, Very difficult]*

Did you find any of the exercises particularly difficult? *[response options: Yes, No]* If “yes”, which were these? *[Free text]*

Were there any exercises that you generally did less of than you were advised to? *[response options: Yes, No]* If “yes”, which were these? *[Free text]*

Which of the following reasons best explain why you generally did less of one or more of the exercises than you were advised to? *[response options: They didn’t seem to help, They were too difficult, They made things worse, They were too painful to do, Other reason(s)…free text]*. Comments *[Free text]*

What could have been done to make you more willing to do the exercises? *[Free text]*

Is there anything that would have improved the experience of participating in this research study? *[Free text]*
